# Crocin Attenuates NLRP3 Inflammasome Activation by Inhibiting Mitochondrial Reactive Oxygen Species and Ameliorates Monosodium Urate-Induced Mouse Peritonitis

**DOI:** 10.3390/cimb45030134

**Published:** 2023-03-03

**Authors:** Ruth Sangare, Iskander Madhi, Ji-Hee Kim, YoungHee Kim

**Affiliations:** Department of Molecular Biology, College of Natural Sciences, Pusan National University, Busan 46241, Republic of Korea

**Keywords:** Crocin, mitochondrial membrane potential, mitochondrial reactive oxygen species, NLRP3 inflammasome, pyroptosis, peritonitis

## Abstract

Crocin is a hydrophilic carotenoid pigment found in the stigma of *Crocus sativus* or the fruit of *Gardenia jasminoides*. In this study, we investigated the effects of Crocin on the activation of the nucleotide-binding oligomerization domain, leucine-rich repeat, and pyrin domain containing 3 (NLRP3) inflammasome in J774A.1 murine macrophage cells and monosodium urate (MSU)-induced peritonitis. Crocin significantly inhibited Nigericin-, adenosine triphosphate (ATP)-, MSU-induced interleukin (IL)-1β secretion, and caspase-1 cleavage without affecting pro-IL-1β and pro-caspase-1. Crocin also suppressed gasdermin-D cleavage and lactate dehydrogenase release and enhanced cell viability, indicating that Crocin reduces pyroptosis. Similar effects were observed in primary mouse macrophages. However, Crocin did not affect poly(dA:dT)-induced absent in melanoma 2 (AIM2) and muramyl dipeptide-induced NLRP1 inflammasomes. Crocin decreased Nigericin-induced oligimerization and the speck formation of apoptosis-associated speck-like protein containing a caspase recruitment domain (ASC). Crocin also dramatically alleviated the ATP-induced production of mitochondrial reactive oxygen species (mtROS). Finally, Crocin ameliorated the MSU-induced production of IL-1β and IL-18 and the recruitment of neutrophils during peritoneal inflammation. These results suggest that Crocin suppresses NLRP3 inflammasome activation by blocking mtROS production and ameliorates MSU-induced mouse peritonitis. Thus, Crocin may have therapeutic potential in various NLRP3 inflammasome-related inflammatory diseases.

## 1. Introduction

Inflammasomes are multiprotein complexes that regulate caspase-1 activation in innate immune cells and induce inflammation against pathogen-associated molecular patterns (PAMPs) and danger-associated molecular patterns (DAMPs). Several types of inflammasomes have been identified, including the nucleotide-binding oligomerization domain, leucine-rich repeat, and pyrin domain containing 1 (NLRP1), NLRP3, NLR Family caspase activation and recruitment domain (CARD) containing 4 (NLRC4), and absent in melanoma 2 (AIM2) [1]. Among these, NLRP3 inflammasome was widely investigated and demonstrated a close relation to various inflammatory diseases.

The NLRP3 inflammasome is formed by the sensor molecule NLRP3, the adaptor protein apoptosis-associated speck-like protein containing a caspase recruitment domain (ASC), and the effector pro-caspase-1. The NLRP3 protein has a pyrin domain (PYD), and the ASC protein consists of PYD and CARD domains. Upon activation, NLRP3 interacts with ASC via PYD, and multiple ASCs combine into a single macromolecular focus, termed an ASC speck. Finally, pro-caspase-1 is recruited to the complex through its CARD domain by ASC to form the NLRP3/ASC/pro-caspase-1 complex [1]. The assembly of the NLRP3 inflammasome leads to self-cleavage and the activation of caspase-1, as well as the subsequent proteolysis of pro-interleukin (IL)-1β and pro-IL-18 into mature and functional IL-1β and IL-18, respectively. Activated caspase-1 also cleaves gasdermin-D (GSDMD), which then forms a membrane pore that facilitates the release of cytosolic contents, such as IL-1β and lactate dehydrogenase (LDH) and induces inflammatory cell death in the process of pyroptosis [2].

NLRP3 inflammasome activation is believed to be mediated by a two-step process: priming (signal 1) and activation (signal 2) [3]. Priming with the Toll-like receptor 4 (TLR4) ligand lipopolysaccharide (LPS) drives the activation of transcription factors, including nuclear factor-kappa B (NF-κB), and upregulates the inflammasome components NLRP3, caspase-1, and pro-IL-1β. Signal 2 (activation) is provided by various PAMPs or DAMPs, such as extracellular adenosine triphosphate (ATP), a bacterial toxin (e.g., Nigericin), and monosodium urate crystals that activate multiple upstream signaling events. These include K^+^ efflux, Ca^2+^ signaling, lysosomal disruption, mitochondrial reactive oxygen species (mtROS) production, and mitochondrial dysfunction [4]. Considering the diversity of activators and the complexity of signaling pathways of the NLRP3 inflammasome, it is not surprising that the NLRP3 inflammasome has been shown to be involved in multiple diseases, such as inflammatory bowel diseases, gout, atherosclerosis, obesity, type 2 diabetes, multiple sclerosis, Alzheimer’s disease, Parkinson’s disease, and cancers [5]. Thus, the NLRP3 inflammasome has been suggested as a potential drug target for neurological, metabolic, and inflammatory diseases [6].

Crocin (crocetin digentiobiose ester) is a hydrophilic carotenoid pigment found in the stigma of *Crocus sativus* (commonly known as Saffron) or the fruit of *Gardenia jasminoides* [7,8]. These plants are primarily used in many kinds of cuisines as flavoring and coloring agents as well as in traditional medicines for the treatment of edema, headache, fever, jaundice, and hypertension [9,10]. A number of studies have demonstrated that Crocin has a wide range of activities, including antioxidant [11,12], anti-inflammatory [13,14], anti-cancer [15], anti-atherosclerotic [16,17], anti-depressant [18], cardioprotective [19], and hepatoprotective effects [20]. Several studies have shown that Crocin inhibits NF-κB activity and suppresses proinflammatory mediators and cytokines [14,21,22]. However, the effects of Crocin on NLRP3 inflammasome activation (signal 2) have not been investigated.

In this study, we demonstrate that Crocin suppresses the signal 2 process of the NLRP3 inflammasome by inhibiting mtROS production and alleviates monosodium urate (MSU)-induced peritonitis.

## 2. Materials and Methods

### 2.1. Materials

Crocin, LPS (phenol extracted from *Salmonella enteritidis*), 3-(4,5-dimethylthiazol-2-yl)-2,5-diphenyltetrazolium bromide (MTT), ATP, uric acid, and other reagents were purchased from Sigma-Aldrich (St. Louis, MO, USA). LDH Detection Kit was purchased from DoGenBio (Seoul, Republic of Korea). Nigericin, poly(dA:dT), and muramyl dipeptide (MDP) were purchased from InvivoGen (San Diego, CA, USA). Antibodies against caspase-1, ASC, and NLRP3 were purchased from Adipogen (San Diego, CA, USA). Antibody against β-actin was purchased from Santa Cruz Biotechnology (Santa Cruz, CA, USA). The antibody against IL-1β was purchased from R&D Systems (Minneapolis, MN, USA). Lipofectamine™ 3000 Transfection Reagent, MitoSOX™ Red, MitoTracker™ Green FM, MitoTracker™ Deep Red FM, Dulbecco’s modified Eagles’ medium (DMEM), fetal bovine serum (FBS) and antibodies against CD11b-APC, and Ly-6G/Ly-6C-PerCP-Cyanine5.5, goat anti-rabbit IgG (H+L) secondary antibody, HRP, and the goat anti-mouse IgG (H+L) secondary antibody, HRP were purchased from Thermo Fisher Scientific (Waltham, MA, USA).

### 2.2. Preparation of MSU Crystals 

MSU crystals were prepared as described previously [23]. Briefly, Uric acid (0.2 g) was dissolved and heated in 40 mL of 0.01 M NaOH, followed by their adjustment to pH 7.2 at 70 °C. The solution was filtered and cooled overnight in a cold room. The crystals that formed were washed and dried. The shape and size of the crystals were examined by a microscope. The MSU crystals were suspended in PBS at a concentration of 20 mg/mL.

### 2.3. Isolation of Mouse Peritoneal Macrophages

Male C57BL/6 mice, purchased from DBL Co., Ltd. (Eumseong, Republic of Korea), were used between 8 and 12 weeks of age (body weight 25–30 g). All animal studies were conducted in accordance with the principles and procedures of the Pusan National University Institutional Animal Care and Use Committee. Thioglycollate (TG) broth (DIFCO, Detroit, MI, USA)-elicited macrophages were harvested 3 days after the intraperitoneal injection of TG (2.5 mL) into mice and were isolated as previously reported [24]. After 4 h of incubation on cell culture plates, adherent macrophages were immediately used for experimentation. 

### 2.4. Cell Culture and Treatment

The J774A.1 murine macrophage cell line was obtained from the Korean Cell Line Bank (Seoul, Republic of Korea). Mouse peritoneal macrophages or J774A.1 cell (5 × 105) was cultured in DMEM supplemented with 10% heat-inactivated FBS at 37 °C in a humidified atmosphere of 5% CO_2_ and 95% air. For NLRP3 inflammasome activation, the cells were primed with 0.1 μg/mL LPS for 3 h and washed twice with PBS. After adding DMEM, cells were pretreated with 125, 250, or 500 μM of Crocin for 3 h and then stimulated with Nigericin (4 μM) for 1 h, ATP (5 mM) for 1 h, or MSU (300 μg/mL) for 6 h. MDP, an NLRP1 inflammasome trigger, or poly(dA:dT), an AIM2 trigger, were transfected with Lipofectamine™ 3000 Transfection Reagent according to the manufacturer’s instructions for 6 h.

### 2.5. Measurement of IL-1β and IL-18 Concentrations

After the treatment of J774A.1 cell and mouse peritoneal macrophages as described in Section 2.4, the culture supernatant was collected, and IL-1β levels were quantified by ELISA (R&D Systems) according to the manufacturer’s instructions. IL-1β and IL-18 levels in peritoneal exudate after peritonitis induction was quantified by ELISA (R&D Systems) according to the manufacturer’s instructions.

### 2.6. Protein Precipitation of Cell Culture Supernatants for IL-1β and Caspase-1

Proteins in J774A.1 cell and peritoneal macrophage culture supernatants were precipitated using ice-cold methanol/chloroform as described previously [25] with some modifications. The supernatant was harvested by centrifugation at 400× *g* for 5 min and precipitated using an equal volume of methanol and 0.25 volumes of chloroform. Samples were vortexed for 10 s and incubated for 5 min on ice. After centrifugation at 1700× *g* for 10 min at 4 °C, the upper phase was removed. The pellet was washed with ice-cold methanol, followed by centrifugation at 1700× *g* for 10 min at 4 °C. The pellet was dried for 30 min, resuspended in a Laemmli sample buffer, and stored at −20 °C.

### 2.7. Western Blot Analysis

The cytosolic extracts were harvested in an ice-cold lysis buffer (1% Triton X-100 and 1% deoxycholate in PBS). Protein content in cytosolic extracts was determined using Bradford reagent (Bio-Rad; Hercules, CA, USA). Proteins in each sample were resolved using 10% SDS-polyacrylamide gel electrophoresis (SDS-PAGE), transferred to a polyvinylidene difluoride (PVDF) membrane, and incubated with the appropriate antibodies. Proteins were visualized using an enhanced chemiluminescence detection system (Amersham Biosciences, Piscataway, NJ, USA) with horseradish peroxidase-conjugated secondary antibodies. An anti-actin antibody was used as a loading control for the cytosolic protein.

### 2.8. LDH Release Assay 

The total and secreted amounts of LDH was determined using an LDH cytotoxicity kit according to the manufacturer’s instruction. Cell supernatants of J774A.1 and mouse peritoneal macrophages were measured at 450 nm (reference wavelength 600–650 nm) using a microplate reader (Bio-Rad).

### 2.9. Cell Viability Assay 

The cell viability was assessed using a microculture colorimetric assay based on MTT. After the treatment of J774A.1 cell and mouse peritoneal macrophage as described in Section 2.4, MTT was added to each well at a final concentration of 50 μg/mL. After incubation for 3 h at 37 °C in 5% CO_2_, the supernatant was removed, and the formazan crystals that were produced in viable cells were solubilized with dimethylsulfoxide (DMSO). The absorbance of each well was measured at 570 nm using a microplate reader (Bio-Rad).

### 2.10. ASC Oligomerization Assay 

ASC oligomerization was determined using disuccinimidyl suberate (DSS) as described previously [26]. Briefly, after the treatment of J774A.1 cell with Crocin and Nigericin as described in Section 2.4, the cells were washed with ice-cold PBS and lysed in an ice-cold buffer (20 mM HEPES-KOH, pH 7.5, 150 mM KCL, 1% NP-40, protease inhibitor cocktail, and 1 mM sodium orthovanadate). The lysate was centrifuged at 5000× *g* for 10 min at 4 °C. The pellets were washed twice in ice-cold PBS, resuspended in 500 μL PBS, and sonicated. The resuspended pellets were treated with 2 mM DSS at 25 °C for 30 min with rotation. Crosslinked pellets were collected by centrifugation at 1700× *g* for 15 min at 4 °C and resuspended in a Laemmli sample buffer for Western blotting.

### 2.11. Immunofluorescence Microscopy

J774A.1 cells were cultured directly on glass coverslips in 24-well plates. After the treatment of J774A.1 cell with Crocin and Nigericin as described in Section 2.4, the cells were fixed with methanol for 10 min at 4 °C and blocked with 5% bovine serum albumin in PBS for 1 h at room temperature (RT). The cells were then stained with an anti-ASC antibody (1:500) for 16 h at 4 °C and secondary fluorescein isothiocyanate-conjugated IgG antibody (1:5000) for 2 h at RT. Nuclei were stained with 1 μg/mL of 4,6-diamidino-2-phenylindole (DAPI) and then analyzed by fluorescence microscopy with an Axioplan 2 microscope (Zeiss, Jena, Germany). The number of ASC specks was counted manually.

### 2.12. Measurement of mtROS 

After treatment of J774A.1 cell with Crocin and ATP as described in Section 2.4, cells were stained with a MitoSOX™ Red mitochondrial superoxide indicator according to the manufacturer’s instructions. Cell fluorescence was monitored by flow cytometry (BD Accuri C6 flow cytometer, BD Biosciences, San Jose, CA, USA). Data were analyzed using the FlowJo software.

### 2.13. MSU-Induced Murine Peritonitis

Male C57BL/6 mice (8–12 weeks old) were intraperitoneally injected with Crocin (125 or 250 mg/kg body weight) or PBS. After 2 h, peritonitis was induced by the intraperitoneal administration of 2 mg of MSU crystals dissolved in 0.5 mL PBS. The control mice were intraperitoneally injected with the same volume of PBS. The mice were euthanized 6 h later. Their peritoneal cavities were washed with 5 mL PBS, and the total number of peritoneal exudate cells (PECs) was counted using a hematocytometer. Lavage fluids were analyzed for IL-1β and IL-18 levels by ELISA. PECs were subjected to staining and flow cytometric analysis. Neutrophil numbers in the PECs were determined by multiplying the total cell numbers by the percentage of (Ly-6G/Ly-6C)+/CD11b+ cells.

### 2.14. Statistical Analysis

All results are expressed as the mean ± SEM. Each experiment was performed in duplicate and repeated at least three times. Statistical analyses were performed using the GraphPad Prism 7 software. All data represented a normal distribution and were statistically analyzed using a one-way analysis of variance (ANOVA), followed by Tukey’s multiple comparisons tests. A value of *p* < 0.05 was considered statistically significant.

## 3. Results

### 3.1. Crocin Suppresses NLRP3 Inflammasome Activation in Nigericin, ATP, or MSU-Stimulated Macrophages 

**Figure 1:**
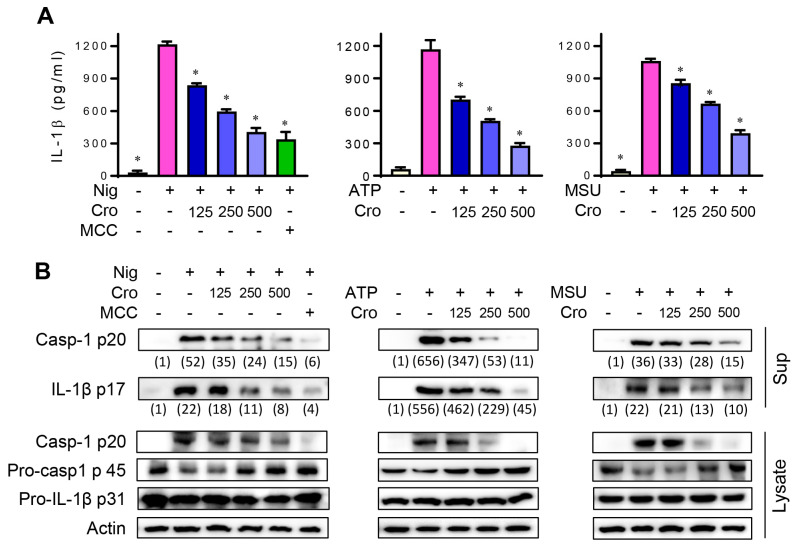
Effects of Crocin on NLRP3 inflammasome. LPS-primed J774A.1 cells were pretreated with various concentrations of Crocin (Cro, μM) for 3 h or 0.1 μM of MCC950 (M) for 1 h, and then incubated with Nigericin (Nig, 4 μM) for 1 h, ATP (5 mM) for 1 h, or MSU (300 μg/mL) for 6 h. (**A**) After incubation, the cell culture supernatant was collected and the amount of IL-1β was measured by ELISA. Data are expressed as the mean ± SEM and represent four experiments. * *p* < 0.05 vs. the group treated with Nigericin, ATP or MSU. (**B**) Simultaneously, the cell culture supernatant was precipitated and the level of cleaved caspase-1 p20 and IL-1β p17 was detected by Western blot. The levels of pro-caspase-1 and pro-IL-1β in cytosolic extract were also detected. The relative intensity of caspase-1 p20 and IL-1β p17 was quantified using image analysis software, ImageJ (http://rsb.info.nih.gov/ij, accessed on 28 February 2023).

We first examined whether Crocin affected IL-1β secretion in response to NLRP3 inflammasome triggers. In the preliminary experiment, we determined the dose of Nigericin, ATP, and MSU in LPS-primed J774A.1 cells by examining the level of cleaved caspase-1 p20 (Supplementary Figure S1). The pretreatment of LPS-primed J774A.1 cells with Crocin significantly inhibited Nigericin-, ATP-, or MSU crystal-induced IL-1β secretion in a dose-dependent manner (Figure 1A). MCC950, a specific inhibitor of NLRP3 inflammasome, was used as a positive control. To further define whether the decreased IL-1β secretion resulted from the inhibitory actions of Crocin on caspase-1 activation and IL-1β maturation, we examined the levels of the active forms of caspase-1 and IL-1β in the cell culture supernatant using Western blotting. As shown in Figure 1B, Crocin markedly reduced the cleaved (active) caspase-1 p20 and active IL-1β p17 levels. These results suggest that Crocin suppresses NLRP3 inflammasome-induced caspase-1 activation and IL-1β maturation. To confirm inhibitory effects of Crocin are not due to its cytotoxicity, we assessed MTT assay in response to Crocin. As shown in Supplementary Figure S2, Crocin with a concentration of up to 500 μM showed no cytotoxicity.

### 3.2. Crocin Inhibits NLRP3 Inflammasome-Induced GSDMD Cleavage and Pyroptosis 

**Figure 2:**
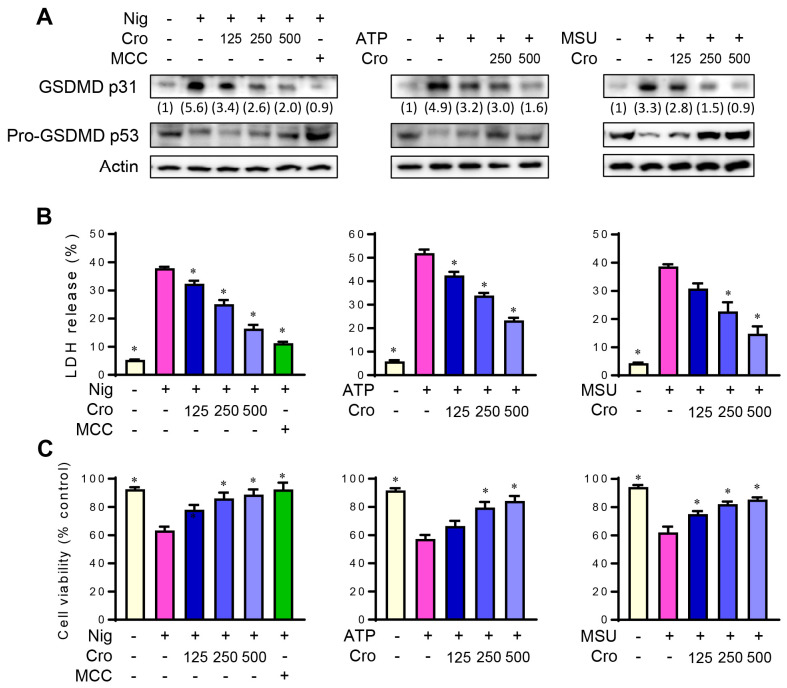
Inhibitory effects of Crocin on GSDMD cleavage, LDH release, and cell viability. LPS-primed J774A.1 cells were pretreated with various concentrations of Crocin (Cro, μM) for 3 h or 0.1 μM of MCC950 (M) for 1 h, and then incubated with Nigericin (Nig, 4 μM) for 1 h, ATP (5 mM) for 1 h, or MSU (300 μg/mL) for 6 h. (**A**) Cleaved GSDMD and pro-GSDMD were detected with cell lysates by Western blotting. Relative intensity of cleaved GSDMD was quantified using image analysis software, ImageJ (http://rsb.info.nih.gov/ij, accessed on 28 February 2023). (**B**) LDH release into the cell culture supernatant was measured. (**C**) Cell viability was assessed by the MTT assay. The data are expressed as the mean ± SEM and represent four experiments. * *p* < 0.05 vs. the group treated with Nigericin, ATP or MSU.

Since the NLRP3 inflammasome triggers GSDMD cleavage and pyroptosis, we examined the effects of Crocin on the GSDMD cleavage using Western blotting. Nigericin, ATP, and MSU crystal increased the cleaved form of GSDMD, while Crocin pretreatment decreased the cleaved forms of GSDMD (Figure 2A). LDH release as a result of inflammasome-induced pyroptosis was also decreased by Crocin pretreatment in a dose-dependent manner (Figure 2B). Consistent with this result, Crocin ameliorated the decreased cell viability in the MTT assay (Figure 2C). These results indicate that Crocin inhibits NLRP3 inflammasome-triggered GSDMD cleavage and pyroptosis.

### 3.3. Crocin Inhibits NLRP3 Inflammasome in Mouse Peritoneal Macrophages

**Figure 3:**
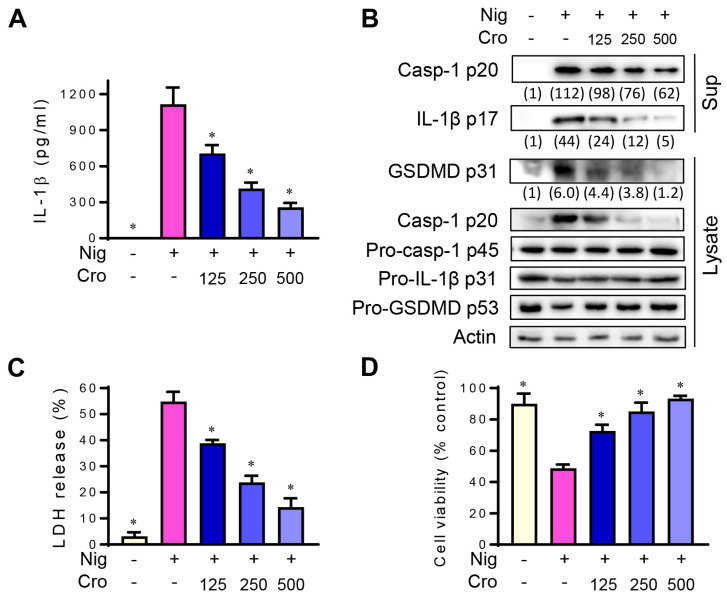
Inhibitory effects of Crocin on NLRP3 inflammasome in mouse peritoneal macrophages. LPS-primed mouse peritoneal macrophages were pretreated with various concentrations of Crocin (Cro, μM) for 3 h, and then incubated with Nigericin (4 μM) for 1 h. (**A**) IL-1β in the cell culture supernatant was measured by ELISA. (**B**) Cleaved caspase-1 p20 and IL-1β p17 in the cell culture supernatant and GSDMD, pro-caspase-1 and pro-IL-1β in cytosolic extract were detected by Western blot. Relative intensity of caspase-1 p20, IL-1β p17, and cleaved GSDMD was quantified using image analysis software, ImageJ (http://rsb.info.nih.gov/ij, accessed on 28 February 2023). LDH release (**C**) and Cell viability (**D**) were assayed as described in Figure 2. The data are expressed as the mean ± SEM and represent three experiments. * *p* < 0.05 vs. the group treated with Nigericin.

To confirm that Crocin suppresses NLRP3 inflammasome not only in the cell line, we isolated mouse peritoneal macrophages and investigated the effects of Crocin on the activation of the NLRP3 inflammasome. Crocin inhibited Nigericin-induced IL-1β secretion (Figure 3A) and the levels of cleaved caspase-1 p20, active IL-1β p17, and cleaved form of GSDMD (Figure 3B). In addition, Nigericin-induced LDH release and cell viability were ameliorated by Crocin treatment (Figure 3C,D). These results suggest that Crocin suppresses the NLRP3 inflammasome, not only in the cell line but also in primary macrophages.

### 3.4. Crocin Does Not Affect NLRP1 or AIM2 Inflammasome

**Figure 4:**
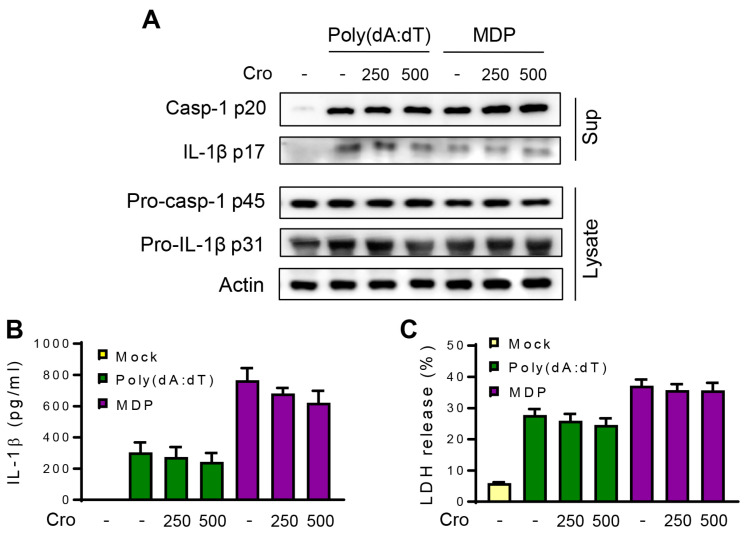
Effects of Crocin on AIM2 and NLRP1 inflammasomes. LPS-primed J774A.1 cells were pretreated with Crocin (Cro, μM) for 3 h and transfected with MDP or poly(dA:dT). (**A**) After 6 h, cleaved caspase-1 p20 and IL-1β p17 were detected in the cell culture supernatant by Western blotting. (**B**) IL-1β in cell culture supernatant was measured by ELISA. (**C**) LDH release was assayed as described in Figure 2. The data are expressed as the mean ± SEM and represent three experiments.

To check whether Crocin inhibits other inflammasomes, such as NLRP1 or AIM2, treatments were performed using MDP (an NLRP1 inflammasome trigger) and poly(dA:dT) (an AIM2 inflammasome trigger). As shown in Figure 4A, the levels of cleaved p20 caspase-1 and active p17 IL-1β in response to MDP or poly(dA:dT) were unchanged by Crocin pretreatment. Moreover, IL-1β secretion and LDH release were not significantly affected by Crocin pretreatment (Figure 4A,C). These results suggest that Crocin does not affect the NLRP1 or AIM2 inflammasomes.

### 3.5. Crocin Suppresses ASC Oligomerization and ASC Speck Formation

**Figure 5:**
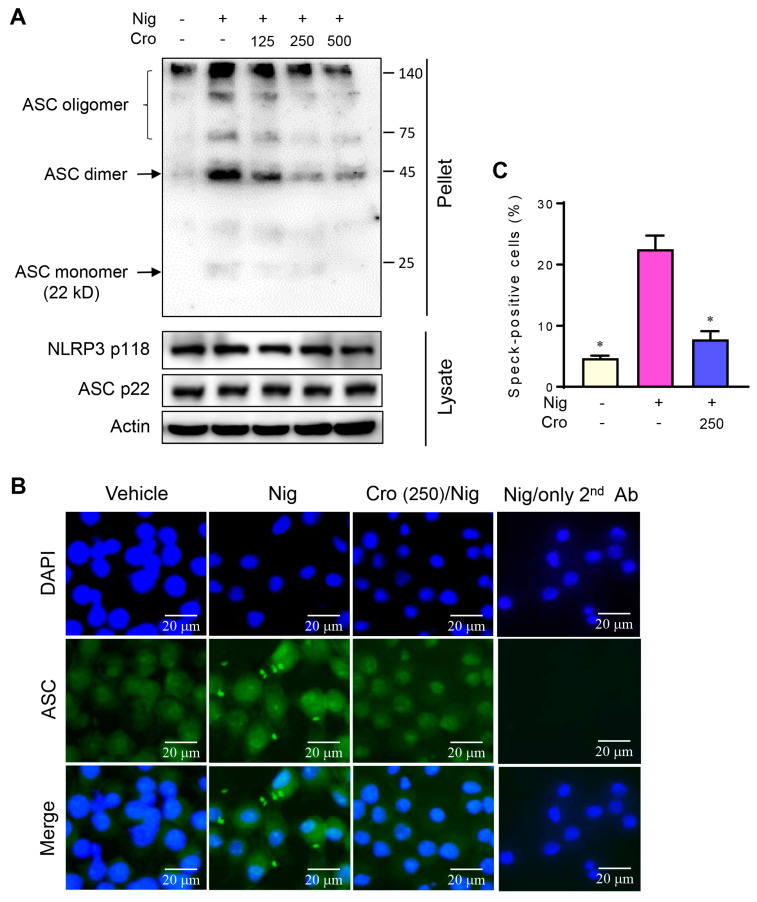
Effects of Crocin on ASC oligomerization and ASC speck formation. LPS-primed J774A.1 cells were pretreated with various concentrations of Crocin for 3 h, and then incubated with Nigericin (4 μM) for 1 h. (**A**) DSS-crosslinked ASC in the NP-40-insoluble pellet was analyzed by Western blot. (**B**) Evidence of ASC specks by fluorescence microscopy. The last column shows a negative control represented by secondary antibody treatment alone. Bars indicate 20 μm. (**C**) Quantification of ASC specks. The data are expressed as the mean ± SEM and represent three experiments. * *p* < 0.05 vs. group treated with Nigericin.

During NLRP3 inflammasome activation, the formation of high molecular weight ASC oligomers is a critical step for subsequent caspase-1 activation. We found that the stimulation of LPS-primed J774A.1 cells with Nigericin promoted the formation of ASC dimers and ASC oligomers in the cell pellets, while pretreatment with Crocin markedly suppressed ASC oligomerization (Figure 5A). Since Crocin did not decrease the total ASC levels in cell lysates, the suppressive effect of Crocin was not due to reduced ASC levels. We also examined the effect of Crocin on ASC speck formation using immunofluorescence microscopy. The speck represents an ASC concentration, which is identified by the ASC antibody. Following Nigericin treatment, ASC multimers were observed in speck-like forms in the cytosol and out of the cell. It can also be observed that the nucleus is smaller following Nigericin treatment, which is consistent with previous reports [27]. Pretreatment with Crocin remarkably reduced the formation of Nigericin-induced ASC specks (Figure 5B,C). These results suggest that Crocin suppresses NLRP3 inflammasome activation by inhibiting the ASC multimer formation.

### 3.6. Crocin Suppresses mtROS Production 

**Figure 6:**
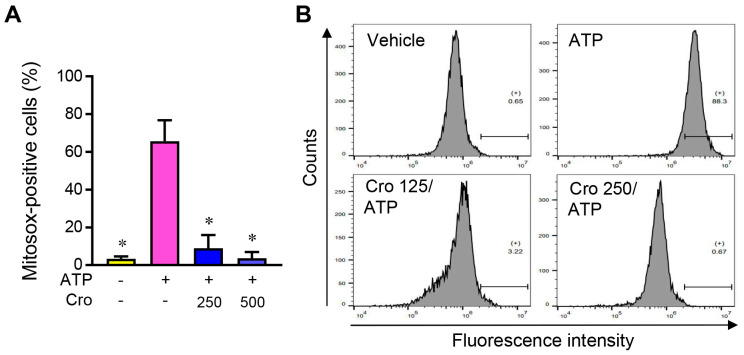
Effects of Crocin on mtROS production and mitochondrial membrane potential. (**A**) LPS-primed J774A.1 cells were pretreated with Crocin (125 μM or 250 μM) for 3 h, and then incubated with ATP (5 mM) for 1 h. Cells were then incubated with MitoSOX for 10 min, and mitochondrial ROS were determined by flow cytometry. (**B**) Representative flow cytometry histograms. The data are expressed as the mean ± SEM and represent three experiments. * *p* < 0.05 vs. group treated with ATP.

To investigate the effect of Crocin on upstream signaling, we examined mtROS levels in LPS-primed J774A.1 cells using a MitoSOX red mitochondrial superoxide indicator. While the level of mitochondrial superoxide increased by the NLRP3 inflammasome trigger ATP, ATP-induced mitochondrial superoxide levels were dramatically diminished by Crocin pretreatment (Figure 6A,B). These results suggest that Crocin inhibits mtROS production during NLRP3 inflammasome activation.

### 3.7. Crocin Inhibits IL-1β and IL-18 Production and Recruitment of Neutrophils in MSU-Induced Peritonitis

**Figure 7:**
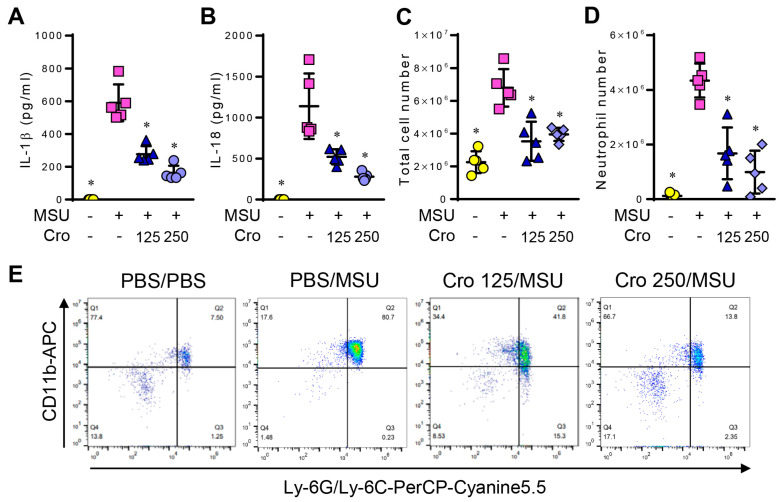
Suppressive effects of Crocin on MSU-induced peritonitis. C57BL/6 mice were intraperitoneally injected with Crocin (125 mg/kg or 250 mg/kg). After 2 h, peritonitis was induced by intraperitoneal injection with MSU (2 mg) for 6 h. (**A**) IL-1β, (**B**) IL-18, (**C**) Total cell number, and (**D**) Neutrophil number from peritoneum exudates. The data are expressed as the mean ± SEM. Each group had five mice. (**E**) Representative flow cytometry histograms. * *p* < 0.05 vs. the group injected with MSU.

The MSU-induced peritonitis model was used to investigate the inhibitory effects of Crocin on NLRP3-mediated inflammation. The intraperitoneal injection of MSU leads to NLRP3 inflammasome-dependent peritonitis, which is characterized by IL-1β and IL-18 production and the neutrophil influx into the peritoneal cavity [28]. Mice were pre-treated with Crocin for 2 h, followed by an intraperitoneal injection of MSU. As expected, Crocin pretreatment considerably decreased MSU-mediated IL-1β and IL-18 production (Figure 7A,B), as well as the total cell number of peritoneal exudates (Figure 7C) and neutrophil recruitment (Figure 7D,E) in mice. These results indicate that Crocin alleviates MSU-mediated peritonitis by inhibiting the NLRP3 inflammasome in vivo.

## 4. Discussion

In this study, we investigated the inhibitory effect of Crocin on NLRP3 inflammasomes in mouse macrophages and a mouse peritonitis model. Crocin pretreatment significantly inhibited the secretion of IL-1β, the cleavage of caspase-1 and IL-1β, and the cleavage of GSDMD and pyroptosis in response to the NLRP3 inflammasome triggers: Nigericin, ATP, and MSU. Crocin could inhibit the NLRP3 inflammasome by reducing the expression of NLRP3 inflammasome components, such as NLRP3 and pro-caspase-1 (at the priming step), as we found in a previous study that Crocin suppresses NF-κB activity [14]. Thus, to minimize the effects on the priming step, we incubated the cells with LPS for 3 h and washed out the remaining LPS. The cells were then treated with Crocin and stimulated with NLRP3 inflammasome triggers. As we expected, pro-caspase-1, pro-IL-1β, and NLRP3 levels were not altered by the NLRP3 inflammasome triggers or by Crocin. Therefore, our experimental system could exclude the effects on the priming step, and our results suggest that Crocin suppresses the NLRP3 inflammasome activation step (signal 2). Similar results were observed in mouse primary macrophages in response to Nigericin treatment. In contrast, Crocin did not inhibit the activation of NLRP1 or AIM2. Thus, our findings suggest that Crocin acts as an NLRP3 inflammasome-specific inhibitor. 

Similar to the in vitro effects, Crocin attenuated the secretion of IL-1β and IL-18 and neutrophil recruitment in an MSU-induced mouse peritonitis model. Abnormal uric acid metabolism results in MSU crystal deposition in the joint and periarticular tissues, which leads to gout: a common form of inflammatory arthritis [28]. The inflammatory response to MSU activates resident macrophages to produce IL-1β and induces neutrophil recruitment [28]. The intraperitoneal injection of MSU crystals in mice has been used as an animal model of gout [28,29,30]. The observation that Crocin alleviated the MSU-induced inflammatory response supports the suggestion that Crocin can ameliorate the symptoms of gout. The finding that Crocin inhibited MSU-induced inflammation indicates that Crocin might relieve NLRP3 inflammasome-related diseases. Zhang et al. [31] recently reported that Crocin alleviates NLRP3 inflammasomes in diabetic kidneys. Our results are consistent with theirs, although they showed that Crocin suppressed the production of IL-1β and IL-18 by inhibiting the expression of the NLRP3 inflammasome. 

ASC oligomerization is considered an essential event for NLRP3 inflammasome activation. Upon activation, NLRP3 inflammasome proteins oligomerize to form scaffolds to aggregate ASC in filaments, resulting in large ASC specks [1]. Many ASC specks appear to be released during pyroptosis [32]. Extracellular and intracellular ASC specks remain active [33]. Thus, extracellular ASC specks can directly activate caspase-1 and can be engulfed by the surrounding cells, resulting in danger signals that activate and trigger an inflammatory cascade and participate in antigen presentation [32,34,35]. Consistent with these reports, we observed ASC specks in the extracellular space (Figure 5B) and cytosol (Figure 5A,B) in response to Nigericin. Extracellular ASC specks have been related to many diseases, including rheumatoid arthritis (RA), systemic lupus erythematosus, gout, psoriasis, allergic rhinitis, chronic obstructive pulmonary disease, Alzheimer’s disease (AD), Parkinson’s disease (PD), cancers, and viral infections [32]. We found that ASC oligomerization was decreased by Crocin pretreatment. Thus, Crocin could have therapeutic potential for the aforementioned diseases, although the remedial effects of Crocin on these diseases have not yet been proven. Supporting this logic, Crocin reportedly has mitigating effects on RA, AD, and PD [36,37,38,39]. 

Signal 2 of NLRP3 inflammasome activation is regulated by various signaling pathways, and there is currently no universal model for its activation [3]. NLRP3 seems to act as a sensor of disrupted homoeostasis, including a perturbed mitochondrial function [40]. Mitochondrial dysfunction induces mtROS generation, which exacerbates mitochondrial damage. Mitochondrial dysfunction or mtROS can trigger NLRP3 activation [41]. In the present study, Crocin attenuated mtROS production induced by an NLRP3 inflammasome trigger. These results suggest that Crocin attenuates the NLRP3 inflammasome by reducing mtROS. This suggestion is supported by reports demonstrating that ROS and oxidized mtDNA activate the NLRP3 inflammasome [41,42,43] and that many chemicals suppress the NLRP3 inflammasome by reducing ROS production [6,44,45,46]. Consistent with our findings, some studies have reported that Crocin alleviates hydrogen peroxide-induced ROS production in the nerve cells [21], dexamethasone-induced ROS production in osteoblasts [47], and methylglyoxal-induced mitochondrial superoxide production in osteoclasts [48]. Recently, it was reported that several mitochondrial components, including cardiolipin and mitochondrial DNA (mtDNA), translocated to the cytosol during mitochondrial damage and triggered NLRP3 activation [41,49,50]. Furthermore, mitochondria-associated endoplasmic reticulum membranes (MAM) facilitated NLRP3 inflammasome assembly [51]. Further studies are needed to define the role of Crocin in signaling pathways, including mtDNA, cardiolipin, and MAM. 

In summary, Crocin suppressed Nigericin-, ATP-, and MSU-induced IL-1β secretion, caspase-1 cleavage, GSDMD cleavage, and pyroptosis in mouse macrophages. Crocin also attenuated ASC oligomerization and mtROS production. In vivo, Crocin treatment alleviated MSU-induced peritonitis. These collective results suggest that Crocin attenuates the NLRP3 inflammasome by inhibiting mtROS production. Therefore, Crocin might have therapeutic potential for anti-inflammatory drugs to treat NLRP3 inflammasome-related diseases. The therapeutic effects of Crocin in inflammatory diseases other than MSU-induced peritonitis should be clarified. 

## Data Availability

All data generated and/or analyzed during this study are included in this published article.

## Supplementary Materials

The following supporting information can be downloaded at: https://www.mdpi.com/article/10.3390/cimb45030134/s1, Figure S1: The cleaved caspase-1 p20 levels in response to NLRP3 inflammasome triggers.; Figure S2: The effect of Crocin on J774A.1 cell viability.
