# Supplementary material for: Crocin Attenuates NLRP3 Inflammasome Activation by Inhibiting Mitochondrial Reactive Oxygen Species and Ameliorates Monosodium Urate-Induced Mouse Peritonitis
Source: Curr Issues Mol Biol. 2023 Mar 3;45(3):2090–104. doi: 10.3390/cimb45030134 (PMC10047758; doi:10.3390/cimb45030134)
Supplement: Supplementary file 1 [file cimb-45-00134-s001.zip › cimb-2212649-supplementary.pdf]

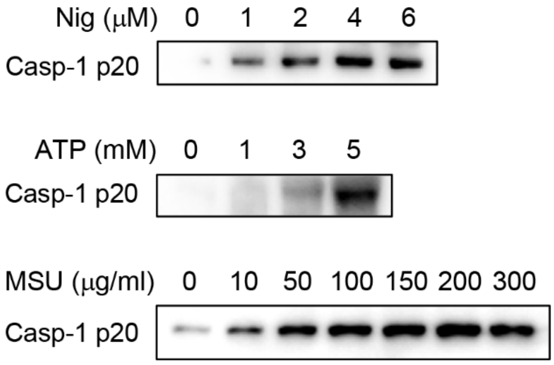


**Supplementary Figure 1.** The cleaved caspase-1 p20 levels in response to NLRP3 inflammasome triggers. LPS-primed J774A.1 cells were treated with the indicated concentration of Nigericin (μM) for 1 h, ATP (mM) for 1 h, and MSU (μg/ml) for 6h. After incubation, cell culture supernatant was precipitated and the level of cleaved caspase-1 p20 was detected by Western blot.

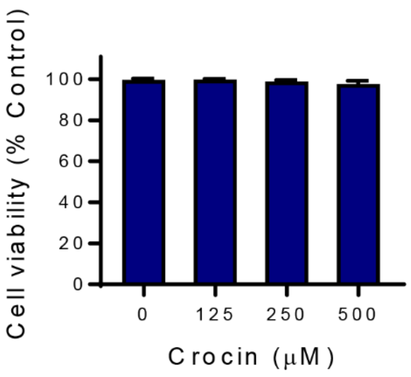


**Supplementary Figure 2.** The effect of Crocin on J774A.1 cell viability. J774A.1 cells were incubated with the indicated concentration of Crocin for 9 h, and cell viability was determined using MTT assay.
